# Identification of Key Receptor Residues Discriminating Human Chorionic Gonadotropin (hCG)- and Luteinizing Hormone (LH)-Specific Signaling

**DOI:** 10.3390/ijms22010151

**Published:** 2020-12-25

**Authors:** Clara Lazzaretti, Valentina Secco, Elia Paradiso, Samantha Sperduti, Claudia Rutz, Annika Kreuchwig, Gerd Krause, Manuela Simoni, Livio Casarini

**Affiliations:** 1 Unit of Endocrinology, Department of Biomedical, Metabolic and Neural Sciences, University of Modena and Reggio Emilia, 41126 Modena, Italy; clara.lazzaretti@unimore.it (C.L.); valentina.secco@unimore.it (V.S.); elia.paradiso@unimore.it (E.P.); samantha.sperduti@unimore.it (S.S.); manuela.simoni@unimore.it (M.S.); 2 International PhD School in Clinical and Experimental Medicine (CEM), University of Modena and Reggio Emilia, 41125 Modena, Italy; 3 Center for Genomic Research, University of Modena and Reggio Emilia, Via G. Campi 287, 41125 Modena, Italy; 4 Leibniz-Forschungsinstitut für Molekulare Pharmakologie (FMP), 13125 Berlin, Germany; RUTZ@fmp-berlin.de (C.R.); kreuchwig@fmp-berlin.de (A.K.); GKRAUSE@fmp-berlin.de (G.K.); 5 Department of Medical Specialties, Azienda Ospedaliero-Universitaria di Modena, 41126 Modena, Italy

**Keywords:** luteinizing hormone (LH), human chorionic gonadotropin (hCG), LH/hCG receptor (LHCGR), mutation, cAMP, ERK1/2

## Abstract

(1) The human luteinizing hormone (LH)/chorionic gonadotropin (hCG) receptor (LHCGR) discriminates its two hormone ligands and differs from the murine receptor (Lhr) in amino acid residues potentially involved in qualitative discerning of LH and hCG. The latter gonadotropin is absent in rodents. The aim of the study is to identify LHCGR residues involved in hCG/LH discrimination. (2) Eight LHCGR cDNAs were developed, carrying “murinizing” mutations on aminoacidic residues assumed to interact specifically with LH, hCG, or both. HEK293 cells expressing a mutant or the wild type receptor were treated with LH or hCG and the kinetics of cyclic adenosine monophosphate (cAMP) and phosphorylated extracellular signal-regulated kinases 1/2 (pERK1/2) activation was analyzed by bioluminescence resonance energy transfer (BRET). (3) Mutations falling within the receptor leucine reach repeat 9 and 10 (LRR9 and LRR10; K225S +T226I and R247T), of the large extracellular binding domain, are linked to loss of hormone-specific induced cAMP increase, as well as hCG-specific pERK1/2 activation, leading to a Lhr-like modulation of the LHCGR-mediated intracellular signaling pattern. These results support the hypothesis that LHCGR LRR domain is the interaction site of the hormone β-L2 loop, which differs between LH and hCG, and might be fundamental for inducing gonadotropin-specific signals. (4) Taken together, these data identify LHCGR key residues likely evolved in the human to discriminate LH/hCG specific binding.

## 1. Introduction

Luteinizing hormone (LH) and chorionic gonadotropin (CG) are two glycoprotein hormones regulating development and reproduction in both sexes. They share a common α and have a unique β subunit responsible of binding the same G protein-coupled receptor (GPCR), the LH/human chorionic gonadotropin (hCG) receptor (LHCGR) [1,2]. In spite of these similarities, the two hormones regulate distinct physiological processes in primates [2]. LH is released by the pituitary gland in a pulsatile fashion and modulates androgen gonadal synthesis and gametogenesis, while hCG is the primate-specific pregnancy hormone fundamental for supporting fetal development and progesterone synthesis [2]. Moreover, hCG differs from LH for slight differences in the aminoacidic sequence on the β subunit, as the β-L2 loop [3,4], the C-terminal extension (CTP), and five additional glycosylation sites [5,6].

In analogy to the crystal structure of the hormone bound extracellular domain of the highly sequence homologues follitropin receptor (FSHR) [7], the LHCGR is characterized by a large extracellular, leucine-rich-repeat domain (LRRD) with hormone binding properties, a hinge region acting as a structural linker involved in the transmembrane helices (TMH)-mediated signal transduction [3,7]. In the human, the receptor is expressed in Leydig cells, where it modulates testosterone synthesis upon LH binding, and, in females of fertile age, in ovarian theca and granulosa cells, as well as in the *corpus luteum*, mediating progesterone and androgen production under the stimulus of LH or hCG [2]. Although differences in LHCGR functioning may occur at the molecular level, between men and women [2,8], the receptor distinguishes the two ligands and mediates hormone-specific intracellular signaling patterns of cyclic adenosine monophosphate (cAMP)/protein kinase A (PKA)-, protein kinase B (AKT)-, and extracellular signal-regulated kinases ½ (ERK1/2)-pathway activation [9,10]. LH is more potent than hCG in activating the phosphorylation of ERK1/2 and AKT, consistently with the requirement of proliferative and survival signals for modulating folliculogenesis. Instead, hCG predominantly triggers steroidogenic events fundamental for supporting pregnancy and delivered mainly through preferential activation of the cAMP/PKA-pathway [10].

The murine receptor (Lhr) is structurally similar to LHCGR and binds both human LH and hCG. This is suggestive of evolutionarily conserved residues regulating the hormone–receptor interaction, leading to the capability of Lhr binding even to primate-specific hormones, i.e., the choriogonadotropin. However, Lhr does not discriminate qualitatively between the LH- and hCG-specific signaling [9]. Therefore, in Lhr-expressing cells, hCG is more potent than LH in activating both the cAMP/PKA- and ERK1/2-pathways (Table 1) [9,10], which is plausibly indicative of different binding affinities [11] and of quantitative, but not qualitative differentiation between the two molecules. These data may be obtained by comparing the hormone 50% (50% effective concentrations, EC_50_) and maximal effective concentrations (EC_max_) (Table 1), as two pharmacological parameters indicative of the amount of molecules, and suggest the existence of LHCGR-specific amino acids, differentiating the LH/hCG dual hormonal system evolved in primates and missing in rodents [12,13].

Computational predictive models identified LHCGR amino acids, falling within the LRRD and the hinge region of the receptor that are interacting with the beta-L2-loop of the hormones and putatively discriminating between LH and hCG [3]. Nevertheless, clear-cut in vitro data confirming these inferences obtained in silico are missing.

**Figure 1:**
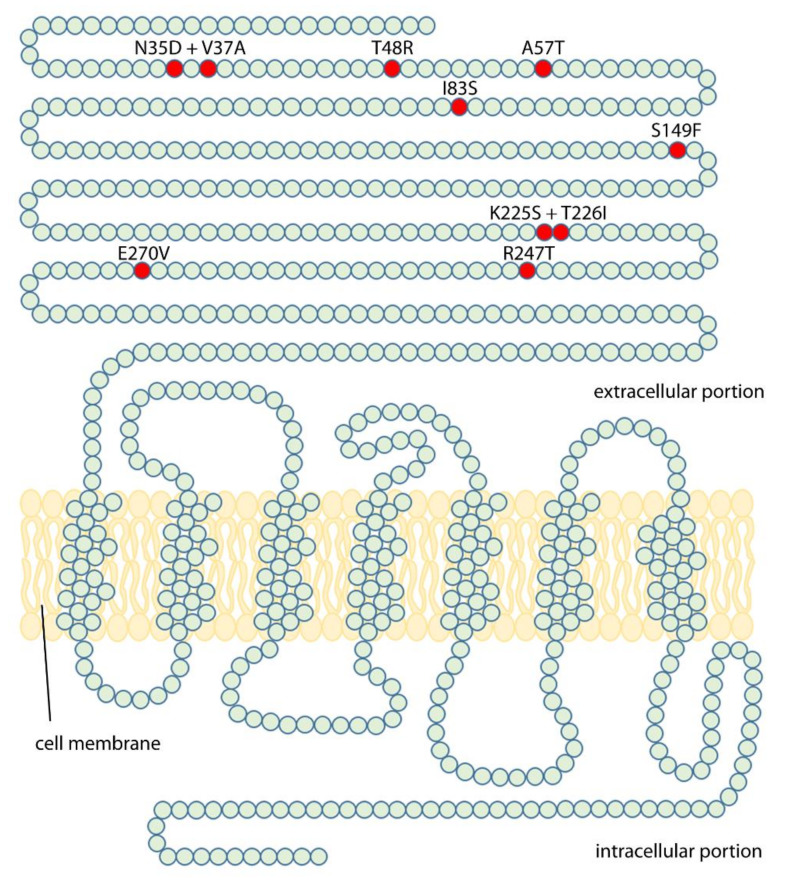
“Murinizing” point mutations induced in the LHCGR protein chain. Amino acid changes were obtained by mutagenesis (see methods) and a total of eight mutant human receptors, carrying mouse luteinizing hormone receptor (mLhr) amino acids in one or two key residues, were obtained.

This study aims to elucidate the origin of the discriminative potential of LHCGR for LH and hCG, in order to understand molecular details of their different pharmacological impact. To this purpose, mutational analysis of LHCGR was performed by introducing “murinizing” amino acids (Figure 1), replacing those residues assumed to be responsible of LH/hCG-specific signal differentiation in humans [14]. Assuming that the two gonadotropins have similar affinity for the wild type and mutant LHCGR, LH-, and hCG-induced cAMP and phosphorylated extracellular signal-regulated kinases 1/2 (pERK1/2) activation was compared for each receptor.

## 2. Results

### 2.1. Analysis of LH- and hCG-Induced cAMP Production

The wild type and mutant LHCGR-mediated cAMP activation was evaluated by bioluminescence resonance energy transfer (BRET), in 1 pM–100 nM LH- and hCG-treated cells transiently co-expressing the cAMP-biosensor and the receptor. cAMP values were indicated as mean ± SEM (Figure 1) and forskolin-treated cells served as a positive control (Figure S1).

**Figure 2:**
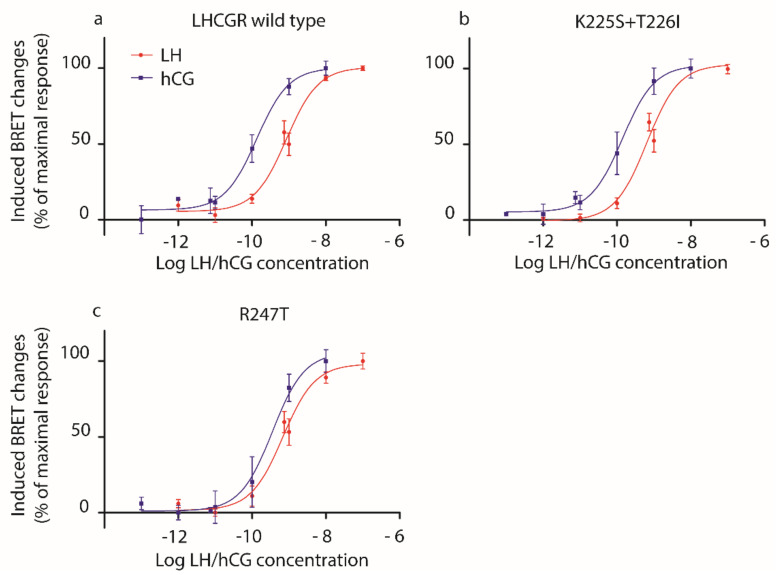
LH- and hCG-induced cAMP response in HEK293 cells overexpressing (**a**) wild type LHCGR, (**b**) the K225S + T226I or (**c**) the R247T mutants. HEK293 cells were transiently transfected with mutant or wild type LHCGR- and CAMYEL-expressing plasmids. Bioluminescence resonance energy transfer (BRET) changes were measured after treating cells 30 min with increasing concentrations of LH or hCG (1 pM–100 nM range) in the presence of 3-isobutil-1-methylxanthine (IBMX). Moreover, 50 µM forskolin served as a positive control. Data were interpolated by non-linear regression and expressed as percentage of the maximal response (means ± SEM; *n* = 4).

None of the mutations impaired completely receptor functionality and the gonadotropin dose-dependent cAMP accumulation was detected in all samples (Figure 2; Figure S2).

We confirmed [10] that hCG is more potent than LH in inducing cAMP increase, in wild type LHCGR-expressing cells, as demonstrated by lower EC_50_ value (Table 2). Similar results were obtained using cells expressing mutant LHCGRs, except for the overall marginal effect mediated by the K225S + T226I and, mostly, the R247T mutants, which are linked to similar LH- and hCG-induced cAMP production (Figure 2; Table 2; Mann–Whitney U test; *p* ≥ 0.05; *n* = 4). The loss of LH/hCG discriminatory potential of these murinized receptors is better indicated by the LH:hCG EC_50_ ratio, which reveals that the choriogonadotropin is only 3.6- and 1.4-fold more potent than LH for the K225S + T226I and R247T mutants, respectively, versus 7.9-fold higher potency of hCG- than LH-induced cAMP response mediated by the wild type LHCGR.

### 2.2. Analysis of LH- and hCG-Induced ERK1/2 Phosphorylation

**Figure 3:**
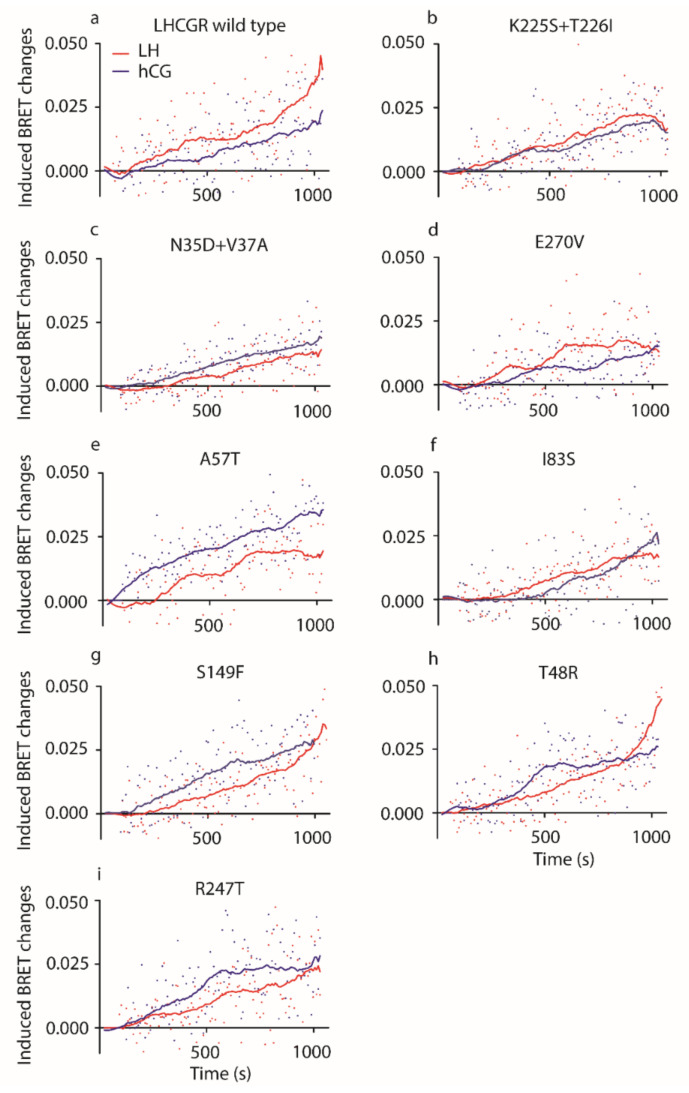
LH- and hCG-induced pERK1/2 activation. In HEK293 cells transiently overexpressing (**a**) the wild type receptor, (**b**) the K225S + T226I, (**c**) the N35D + V37A, (**d**) the E270V, (**e**) A57T, (**f**) I83S, (**g**) the S149F, (**h**) the T48R or (**i**) the R247T mutants, the kinetics of ERK1/2 phosphorylation was analyzed. Induced BRET changes obtained thanks to the interaction between the REV BRET-biosensor and pERK1/2 were measured over 1200 s upon administration of 100 pM LH or hCG. Data from LH/hCG-treated cells were represented by locally weighted scatterplot smoothing (LOWESS) of the induced BRET changes. All the LH- vs hCG-induced kinetics of ERK1/2 phosphorylations are different, except those mediated by the K225S + T226I and I83S mutants (Kruskal–Wallis test and Dunn’s Multiple Comparison post-test; *p* ≥ 0.05; each point is the mean value from eight independent experimental replicates).

The impact of “murinizing” LHCGR mutations on pERK1/2 activation was assessed in transfected HEK293 cells treated by 100 pM LH or hCG, as the hormone concentration maximally activating pERK1/2 and resulting in significantly higher LH- than hCG-induced response [10]. The kinetics of pERK/2 activation was evaluated over 20 min and 1 ng/µL of PMA served as a positive control (Figure S3). Results are indicated in an x-y graph by locally weighted scatterplot smoothing (LOWESS) curves of the induced BRET changes over the baseline (Figure 3).

All mutant LHCGRs mediate hormone-induced ERK1/2 phosphorylation, although some point mutations modulated pERK1/2 activation (Figure 3). Different activation kinetics were found upon cell treatment by LH or hCG (Kruskal–Wallis test and Dunn’s multiple comparison post-test; *p* < 0.05; *n* = 8), except for those mediated by the K225S + T226I and I83S mutant LHCGRs (Kruskal–Wallis test and Dunn’s Multiple Comparison post-test; *p* ≥ 0.05; *n* = 8; means).

Since previous analyses demonstrated that LH is more potent than hCG in activating pERK1/2 [9,10,15], areas under the curve (AUCs) obtained from LOWESS were compared (Table 3). LH AUC is significantly larger than that of hCG calculated from the kinetic curves mediated by wild type receptor (Table 3; two-way ANOVA and Sidak’s correction for multiple test; means ± SEM, relative units; *p* < 0.05; *n* = 8) confirming the preferential activation of proliferative signal operated by the lutropin in comparison to hCG [10]. Moreover, hCG AUC is larger than that of LH when LHCGR carries the A57T and R247T mutations (Table 3; two-way ANOVA and Sidak’s correction for multiple test; means ± SEM, relative units; *p* < 0.05; *n* = 8), revealing that these amino acid changes reverse the relationship between LH and hCG potencies in activating pERK1/2. Finally, we found that the activation of pERK1/2 mediated by N35D + V37A, A57T, and I83S LHCGRs resulted in overall different AUCs than those obtained after treatment of wild type LHCGR-expressing cells (Table 3; two-way ANOVA and Sidak’s correction for multiple test; means ± SEM, relative units; *p* < 0.05; *n* = 8), indicating that these mutations are linked to altered response to gonadotropins.

## 3. Discussion

We identified the LHCGR hot spots fundamental for discriminating between LH and hCG, transforming the human receptor in a mouse-like receptor upon induction of point mutations in a very small number of key residues. Therefore, this study provides new molecular insights on how the LRRD portion of the human receptor differentiates the two ligands, modulating hormone-specific signaling and physiology. The amino acid residues involved in these functions are barely known and were previously investigated by in silico approaches [3,16], while data obtained in vitro were missing. Amino acids crucial for recognizing the dual primate-specific ligand system were predicted by structural modelling of the human and murine receptors to fall within the extracellular hormone binding site of the human hormones at the LRRD and hinge region of the receptor [3]. The models served for generating “murinized” LHCGR molecules replacing human amino acids by the corresponding murine Lhr residues (Figure 1; Table 4 and Table 5). K225S + T226I (on LRR-9) and, especially, the R247T (on LRR-10) mutation induced the loss of hormone-specific differences of cAMP production mediated by LHCGR, while A57T (cysteine-rich region) and R247T mutations increased the hCG-specific potency in activating pERK1/2, which become even higher than that of LH, resulting in “murinization” of the human receptor functioning. Indeed, Lhr does not qualitatively discriminate between the two hormones [9], consistently with the lack of hCG in rodents. This suggests that the Lhr lacks of residues, such as the charged amino acids K225 on LRR-9 and E270 on LRR-11, discriminating the LH- and hCG-specific activity in LHCGR. In particular, the R247 amino acid falling within the LRR-10 could be one of the main human receptor hot spot for differentiating the action of the two hormones, since the arginine change to threonine at position 247 is linked to increased hCG-induced phosphorylation of ERK1/2 together with dramatic loss of hormone potency for cAMP activation. Other LHCGR mutations, i.e., N35D + V37A, A57T and I83S, perturbed the receptor-mediated activation of pERK1/2 without no substantial differences between LH- and hCG-induced responses. These conclusions are supported by LH:hCG concentration ratios calculated for cAMP and pERK1/2 activation (Table 4). Indeed, certain LHCGR spots play a key role in discriminating hormone-specific activities and the change of these amino acids to the corresponding mouse residue results in the “murinization” of the response to gonadotropins.

**Figure 4:**
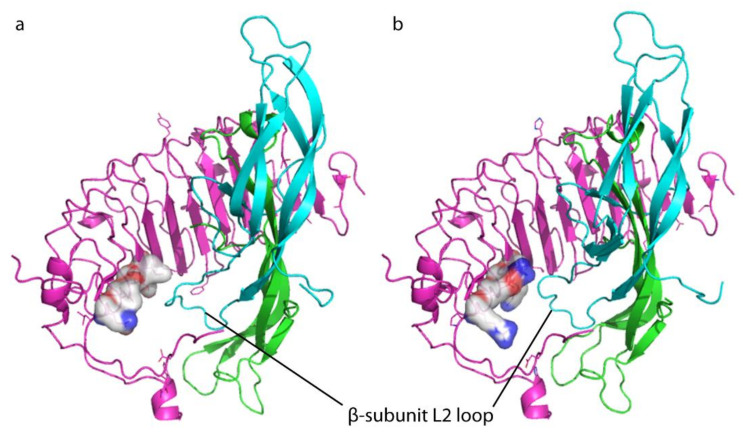
Comparison between mouse and human receptor extracellular leucine-rich-repeat domain (LRRD) bound to the heterodimeric hormone (α and β subunit). (**a**) Images were obtained using hCG sequence as a template for the hormone, as previously described [3], and the α and β subunits are indicated with the colors green and light blue, respectively. Receptor LRRD is pink, while the murine and human LRR11 residue at positions 247, as well as the adjacent residues at position 225 and 270, are indicated by bubbles. mLhr LRRD- and (**b**) LHCGR LRRD-hCG complexes. The hormone β-L2 loop might potentially interact with these different, key residues of rodent and human receptors.

Predictive hormone-receptor interaction models indicate that the β-L2 loop of the two hormones likely interacts with the LRRD of the receptor [3], and results obtained inducing the double K225S + T226I mutation, falling within the ninth LRR (LRR9), support this hypothesis. Indeed, the “murinization” of this spot resulted in the loss of different LH- and hCG-induced cAMP and pERK1/2 responses. Similar effects on cAMP production were obtained by the R247T mutation, which shifts the preferential hCG activity towards pERK1/2-, instead of cAMP/PKA pathway activation. These findings might be the result of different binding affinities between LH and hCG for LHCGR, as previous experiments using rodent receptors suggested [11]. However, these data should be confirmed by displacement experiments performed in cells expressing the human receptor, using both radio-labeled gonadotropins. To date, only displacement data obtained using [^125^I]hCG were available [14,17], while similar experiments using [^125^I]LH were never performed. This is an unanswered question of LH/hCG molecular biology, which is limited by the use of radioactive compounds and technical difficulties in labelling glycosylated hormones. In any case, it may be supposed that the larger and sterically bulkier side chains of LHCGR can apparently discriminate between the different β-L2 loop conformations of the two hormones, while the smaller side chains of the murine receptor are not able to do so (Figure 4). The E270V mutation, falling within the LRR11 as part of the hinge region, is linked to a drop of LH, but not of hCG potency in increasing the intracellular cAMP, in spite of no effects on pERK1/2 activation compared to the wild type receptor and suggesting it could be a site relevant for the functioning of the hinge region. Previous data demonstrated that deletions carried by the LHCGR hinge region impaired the steroidogenic potential of LH, while hCG remains functional [17,18].

It was demonstrated that gonadotropin-induced intracellular signals may vary according to LHCGR (and FSHR) coupling to G proteins and/or β-arrestins, as a feature depending on the expression levels [19,20,21]. However, the different nature of LH/hCG interaction with the LHCGR [3,16] is the origin of downstream hormone-specific signals, likely initiating with different Gs and Gq protein activation [22] and β-arrestin recruitment [15], impacting the intracellular signaling pattern [2]. The different interaction of LH and hCG with the receptor were firstly suggested by a clinical case report. The androgenisation of an adult male patient with hypergonadotropic hypogonadism, lack of secondary sexual characteristic and failure of testosterone production was partially recovered by injections of exogenous hCG, while the high endogenous LH had no effects [18]. This patient carried a DNA sequence deletion affecting the conformational structure of LHCGR, impairing LH- but not hCG-mediated signaling, although maintaining the capability of bind both hormones [16,17]. Further findings demonstrated that the treatment of human primary granulosa cells by LH predominantly activates the ERK1/2- and AKT-pathways, while hCG has higher potency than the lutropin in increasing the intracellular cAMP in vitro [10]. These peculiarities are reflected in different life and death signals modulated by the two molecules in primates, and consisting in LH-dependent proliferative and anti-apoptotic effects, as well as in steroidogenic and pro-apoptotic events mediated by hCG in vitro [23]. Non-primate mammals have no choriogonadotropins and the regulation of gametogenesis, androgen synthesis and pregnancy is undertaken by molecules encoded from the *lhb* gene. Reflecting the presence of a unique Lhr ligand during the evolution and a relatively high sequence similarity to LHCGR, the rodent receptor may bind human LH and hCG but does not qualitatively differentiate the cAMP/PKA-, ERK1/2, and AKT activation, resulting in similar LH- and hCG-induced steroid synthesis [9].

The impact of different LH- and hCG-induced signals would impact the ovarian follicle growth and quality, since the addition of these molecules to FSH in women undergoing assisted reproduction improved different clinical outcomes [24]. Taken together, these data are suggestive of the existence of LHCGR residues deputed to LH and hCG discrimination evolved in primates, allowing to separate their endocrine signals and optimizing follicular development and pregnancy support. We could suppose that these residues, unique of LHCGR, are the result of receptor-ligands co-evolution [25,26], driving the regulation of endocrine reproductive functions [27]. It was hypothesized that hCG appeared in primates for enhancing the trophoblast invasion of maternal tissues, as a requisite for providing proper energetic support to the fetal brain development [28]. These prerogatives are lacking in mice and other mammals, where pregestational effects relies on the LH action mediated by a receptor evolved for binding this unique ligand.

## 4. Materials and Methods

### 4.1. Recombinant Hormones

All in-vitro experiments were performed using LHCGR-overexpressing transfected HEK293 cells treated by recombinant human LH and hCG (Luveris and Ovitrelle; Merck KGaA, Darmstadt, Germany).

### 4.2. HEK293 Cell Line Cultures and Handling

HEK293 cells were maintained in Dulbecco’s Modified Eagles Medium (DMEM) with the addiction of 10% fetal bovine serum (FBS), 100 IU/mL penicillin, 50 µg/mL streptomycin, and 2 mM L-glutamine (all from Sigma-Aldrich, St. Louis, MO, USA). The cells were cultured in Petri dishes at 37 °C and 5% CO_2_ in an incubator and separated by 0.5% trypsin and 0.2% EDTA diluted 1:10 in phosphate buffered saline (PBS; Sigma-Aldrich) when achieved 70% confluence. 2-days transiently transfected cells were treated by gonadotropins after over-night starvation. Intracellular cAMP increase was evaluated as a measure of the steroidogenic pathway activation, while the phosphorylation of ERK1/2 was indicative of proliferative signals. Data were collected by bioluminescence resonance energy transfer (BRET).

### 4.3. Plasmid Vectors of Mutant and Wild Type LHCGR

**Figure 5:**
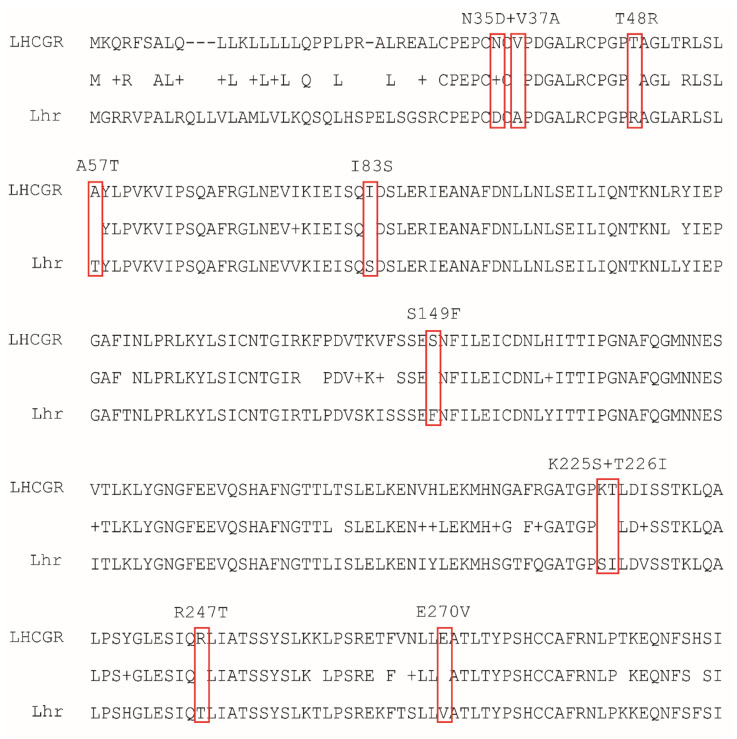
Alignment of LHCGR and Lhr aminoacidic extracellular domain sequences (1–300 aa), using the single-letter code for each amino acid. LHCGR amino acids in positions 35 and 37, 48, 57, 83, 149, 225, and 226, 247, 270 were substituted by mutagenesis with the corresponding murine amino acid (red boxes), as these are residues possibly involved in LH-hCG recognition [14].

The cDNA encoding the wild type LHCGR were inserted into a pcDNA3.1 plasmid under the transcriptional control of the human cytomegalovirus promoter. Mutant “murinized” *LHCGRs* were obtained by mutagenesis of the wild type template using specific primer sequences (Table 5). These new, mutant nucleotides introduced missense mutations corresponding to murine Lhr residues, replacing those of the human receptor at the same position of the protein sequence (Figure 5), and were predicted not to change the tertiary receptor structure using the SWISS-MODEL server (https://swissmodel.expasy.org). Given these characteristics, mutant receptors should not have differential expression than the wild type LHCGR. In fact, raw data of LH and hCG dose–response curves revealed that each receptor allows to achieve similar cAMP plateau levels, suggesting they are effectively and similarly expressed (Figure S4).

### 4.4. Transfection Protocols

A previously described transfection protocol was used [15]. For BRET measurements of cAMP, 3 × 10⁴ cells were seeded in a 96-well plate and transiently transfected with 50 ng/well of cAMP biosensor (CAMYEL)-expressing pcDNA3.1 vector (OriGene Technologies, Rockville, MD, USA) carrying the yFP-RAPGEF3-rluc8 fusion sequence [29], 100 ng of mutant or wild type LHCGR-expressing vector, and 0.5 µL of Metafectene PRO reagent per well (Biontex, München, Germany), according to the manufacturer’s instructions.

Measurement of pERK1/2 activation by BRET were performed by seeding cells at a density of 5 × 10^3^ per well, of a 96-well plate, and transiently transfected with 20 ng/well of pERK1/2 biosensor. This compound was previously described [30] and consists in a rluc8-ERK-substrate-Venus (REV)-expressing vector containing the pRK5-Rluc8-EKAR_cyto_-Venus fusion gene, 100 ng of mutant or wild type LHCGR-expressing vector and 0.5 µL of Metafectene PRO reagent per well (Biontex), according to the manufacturer’s instructions. Cells were then incubated at 37 °C and 5% CO_2_ for 48 h to allow the expression of the receptors and the biosensors.

### 4.5. cAMP Measurement by BRET

Transiently transfected cells were washed and preincubated at 37 °C and 5% CO_2_ for 20 min in 40 µL of phosphate buffered saline (PBS) added with 1 mM HEPES and 500 µM of the phosphodiesterase inhibitor 3-isobutil-1-methylxanthine (IBMX). Then, cells were washed by PBS and maintained for 30 min at 37 °C and 5% CO_2_ in 50 µL of PBS and 1 mM HEPES, in the presence or absence of increasing doses of LH or hCG (1 pM–100 nM range) [10]. Cells treated with 50 µM forskolin (Sigma-Aldrich) served as positive controls [31]. Bioluminescence resonance energy transfer (BRET) measurements were performed upon addition of 10 µL of 5 µM coelenterazine H (Interchim, Montluçon, France). Signals emitted by donor and acceptor tags were detected by the CLARIOstar microplate reader (BMG Labtech, Ortenberg, Germany) at wavelengths of 480 ± 20 and 540 ± 20 nm, respectively, and represented as a ratio (BRET changes) [32].

### 4.6. Evaluation of pERK1/2 Activation by BRET

Forty-eight-hour transiently transfected cells were washed and maintained for 1 h in 100 µL of serum-free media at 37 °C and 5% CO_2_. Cells were washed with PBS and incubated for 20 min in 40 µL PBS added with 1 mM HEPES. Upon automatic injection of 10 µL of 5 µM coelenterazine H and 1 mM HEPES diluted in 20 µL of PBS, in the presence or in the absence of 100 pM LH or hCG, as the hormone concentration maximally activating pERK1/2 [10]. BRET signals were detected using a CLARIOstar microplate reader. Cells treated by phorbol-12-myristate-13-acetate served as positive controls [33]. Light emissions at 480 and 540 ± 20 nm were detected over 17 min, at intervals of 1.76 s, and represented as acceptor/donor ratio.

### 4.7. Statistics

cAMP data were represented as BRET changes over the baseline and values are mean ± standard error of mean (SEM) in a graph with a logarithmic X-axis. Dose–response curves were obtained by data interpolation using non-linear regression. The 50% effective concentrations (EC_50_) were extrapolated and compared using the Mann–Whitney U test, after testing the departure from normality using the D’Agostino and Pearson test. pERK1/2 kinetic curves were represented as locally weighted scatterplot smoothing (LOWESS) of the induced BRET changes over the baseline. Multiple comparison between LOWESS functions of pERK1/2 activation kinetic curves were performed using the Kruskal–Wallis test and Dunn’s Multiple Comparison post-test, after testing the departure from normality using the D’Agostino and Pearson test. The area under the curve (AUC) was also calculated from the LOWESS, considering the time-window of 10–15 min as that displaying optimal pERK1/2 activation [10]. AUCs were then compared using the Mann–Whitney U test, after the exclusion of outliers calculated by the Grubb’s test. Statistical analysis was performed using GraphPad Prism software (GraphPad Software Inc., San Diego, CA, USA) and differences were considered as significant for *p* < 0.05.

## Figures and Tables

**Table 1:** Luteinizing hormone (LH) and human chorionic gonadotropin (hCG) 50% effective concentrations (EC_50_), and maximal effective concentrations (EC_max_) for cyclic adenosine monophosphate (cAMP) and phosphorylated extracellular signal-regulated kinases 1/2 (pERK1/2) activation (pM; means ± SEM; *n* = 3–5). LHCGR = LH/hCG receptor; Lhr = the murine receptor; Ref. = reference.

Receptor.	Cell Type	Hormone	cAMP EC_50_	pERK1/2 EC_max_	cAMP:pERK1/2 Ratio	Ref.
LHCGR	Human Granulosa	LH	530 ± 51	100	5:1	[10]
		hCG	107 ± 14	100	1:1	
Lhr	Mouse Leydig	LH	192 ± 54	100	2:1	[9]
		hCG	18 ± 10	10	2:1	

**Table 2:** LH and hCG EC_50_ extrapolated from dose-response curves after cAMP measurements.

LHCGR Mutation	LH EC_50_ (pM)	hCG EC_50_ (pM)	*p* Value
K225S + T226I	697.5 ± 165.9	193.5 ± 100.2	0.0571
N35D + V37A	595.4 ± 154.8	94.49 ± 3.38	0.0286
E270V	2806.0 ± 1393.0	248.4 ± 40.65	0.0286
A57T	702.6 ± 222.9	152.8 ± 33.91	0.0286
I83S	711.9 ± 187.7	127.2 ± 6.28	0.0286
S149F	755.8 ± 173.3	111.0 ± 23.66	0.0286
T48R	1207.0 ± 389.1	130.5 ± 19.58	0.0286
R247T	556.5 ± 31.63	386.2 ± 132.0	0.6286
Wild type	1111.0 ± 378.2	142.8 ± 30.85	0.0286

(Mann–Whitney U test; *p* < 0.05; means ± SEM; *n* = 4).

**Table 3:** LH and hCG area under the curve (AUC) calculated from ERK1/2 activation kinetic curves (two-way ANOVA and Sidak’s correction for multiple test; means ± SEM, relative units; *n* = 8). NA = not assessed; ns = not significantly different.

LHCGR Mutation	LH AUC	hCG AUC	*p* Value (LH vs. hCG)	*p* Value (vs. Wild Type)
K225S + T226I	11.40 ± 1.30	9.47 ± 1.70	ns	ns
N35D + V37A	5.48 ± 0.63	8.19 ± 1.35	ns	<0.0001
E270V	10.93 ± 1.14	7.18 ± 0.93	ns	ns
A57T	10.89 ± 3.19	20.50 ± 4.86	<0.0001	<0.0001
I83S	8.03 ± 1.85	6.65 ± 1.08	ns	<0.0001
S149F	10.40 ± 1.64	13.91 ± 3.07	ns	ns
T48R	12.28 ± 1.56	13.44 ± 3.94	ns	ns
R247T	10.53 ± 1.07	15.44 ± 2.36	<0.01	ns
Wild type	13.92 ± 1.60	7.98 ± 0.80	<0.0001	NA

**Table 4:** LH:hCG ratio from E50 for cAMP activation, and AUC of pERK1/2 kinetics, mediated by LHCGR. Data were calculated from Table 1 and Table 2 of the present study where indicated.

LHCGR Mutation.	LH:hCG EC_50_ Ratio for cAMP Activation	LH:hCG AUC Ratio for pERK1/2 Activation	Reference
K225S + T226I	3.6	1.2	Present study
N35D + V37A	6.3	0.7	Present study
E270V	11.3	1.5	Present study
A57T	4.6	0.5	Present study
I83S	5.6	1.2	Present study
S149F	6.8	0.7	Present study
T48R	9.2	0.9	Present study
R247T	1.4	0.7	Present study
Wild type	7.8	1.7	Present study
Wild type	5.0	1.0 (calculated on EC_max_)	[10]
Lhr	10.7	10.0 (calculated on EC_max_)	[9]

**Table 5:** Murinizing mutations introduced in the human LHCGR. Nucleotides inducing the amino acid changes are indicated in bold and underlined.

Amino Acid Change	Forward Primer Sequence	Reverse Primer Sequence	Structural Localization
K225S + T226I	CCACAGGGCCGA**GTATCT**TGGATATTTC	GAAATATCCAAGATACTCGGCCCTGTGG	LRR-9
N35D + V37A	GAGCCCTGC**GAC**TGC**GCG**CCCGACGGCG	CGCCGTCGGGCGCGCAGTCGCAGGGCTC	Cystein-rich region 1
E270V	CAATCTCCTG**GTG**GCCACGTTGAC	GTCAACGTGGCCACCAGGAGATTG	Hinge region (LRR-11)
A57T	GACTATCACT**TAC**CTACCTCCCTG	CAGGGAGGTAGGTAAGTGATAGTC	LRR-2
I83S	GAAATCTCTCAG**AGT**GATTCCCTGG	CCAGGGAATCACTCTGAGAGATTTC	LRR-3
S149F	CCTCTGAA**TTC**AATTTCATTCTGG	CCAGAATGAAATTGAATTCAGAGG	LRR-5
T48R	GCCCCGGCCCC**AGG**GCCGGTCTC	GAGACCGGCCCTGGGGCCGGGGC	Cystein-rich region 1
R247T	GTCCATTCAG**ACG**CTAATTGCCACG	CGTGGCAATTAGCGTCTGAATGGAC	LRR-10

## Data Availability

The data presented in this study are available in the article and supplementary materials.

## Supplementary Materials

The supplementary materials are available online at https://www.mdpi.com/1422-0067/22/1/151/s1.

## Abbreviations

LH.	Luteinizing hormone
hCG	Human chorionic gonadotropin
GPCR	G protein-coupled receptor
LHCGR	Human LH/hCG receptor
Lhr	Murine luteinizing-hormone receptor
CTP	C-terminal extension
LRRD	Leucine-rich-repeat domain
cAMP	Cyclic adenosine monophosphate
PKA	Protein kinase A
AKT	Protein kinase B
ERK1/2	Extracellular signal-regulated kinases 1/2
HEK293	Human Embryonic kidney 293
DMEM	Dulbecco’s Modified Eagles Medium
FBS	fetal bovine serum
EDTA	Ethylenediaminetetraacetic acid
PBS	Phosphate buffered saline
BRET	Bioluminescence resonance energy transfer
pERK1/2	Phosphorylated extracellular signal-regulated kinases 1/2
HEPES	4-(2-hydroxyethyl)-1-piperazineethanesulfonic acid
IBMX	Phosphodiesterase inhibitor 3-isobutil-1-methylxanthine
EC50	50% effective concentrations
LOWESS	Locally weighted scatterplot smoothing
AUC	Area under the curve
SEM	Standard error of the mean
PMA	Phorbol 12-myristate 13-acetate
