# Supplementary material for: Identification of Key Receptor Residues Discriminating Human Chorionic Gonadotropin (hCG)- and Luteinizing Hormone (LH)-Specific Signaling
Source: Int J Mol Sci. 2020 Dec 25;22(1):151. doi: 10.3390/ijms22010151 (PMC7794846; doi:10.3390/ijms22010151)
Supplement: Supplementary file 1 [file ijms-22-00151-s001.zip › Supplementary figure captions.docx]

Supplementary figure captions

**Supplementary figure 1**. Positive controls of cAMP increase in wild-type and mutant LHCGR-expressing HEK293 cells. Samples were treated 30 min with 50 µM forskolin, in the presence of 500 µM IBMX. cAMP was measured by BRET and represented as induced changes of BRET signals over unstimulated cells. A total of three independent experiments were performed and indicated by dots, while lines indicate mean values.

**Supplementary figure 2**. LH- and hCG-induced cAMP response in HEK293 cells overexpressing mutant LHCGRs. (a) N35D + V37A; (b) the E270V (c); A57T; (d) I83S; (e) S149F; (f) T48R. HEK293 cells were transiently transfected with mutant or wild type LHCGR- and CAMYEL-expressing plasmids. BRET changes were measured after treating cells 30 min with increasing concentrations of LH or hCG (1 pM - 100 nM range) in the presence of IBMX. 50 µM forskolin served as a positive control. Data were interpolated by non-linear regression and expressed as percentage of the maximal response (means ± SEM; n=4).

**Supplementary figure 3**. PMA AUC calculated from ERK1/2 activation kinetic curves. Signals were detected over 1200 s in a total of four independent experiments, represented by dots, while lines indicate mean values.

**Supplementary figure 4**. Raw data of LH- and hCG-induced cAMP response in HEK293 cells overexpressing mutant LHCGRs. (a) LH-induced cAMP levels; (b) hCG-induced cAMP levels. Cells were transiently transfected with mutant or wild type LHCGR- and CAMYEL-expressing plasmids. BRET changes were measured after treating cells 30 min with 1 pM - 100 nM LH or hCG and data were connected by lines (means ± SEM; n=4). Statistical analysis revealed that *plateau* levels are similar (Kruskal-Wallis test and Dunn’s multiple comparison post-test; p<0.05). Normalized and log-transformed curves are showed in the figure 2 and supplementary figure 2.
