# Supplementary figures and images for: Identification of Key Receptor Residues Discriminating Human Chorionic Gonadotropin (hCG)- and Luteinizing Hormone (LH)-Specific Signaling
Source: Int J Mol Sci. 2020 Dec 25;22(1):151. doi: 10.3390/ijms22010151 (PMC7794846; doi:10.3390/ijms22010151)

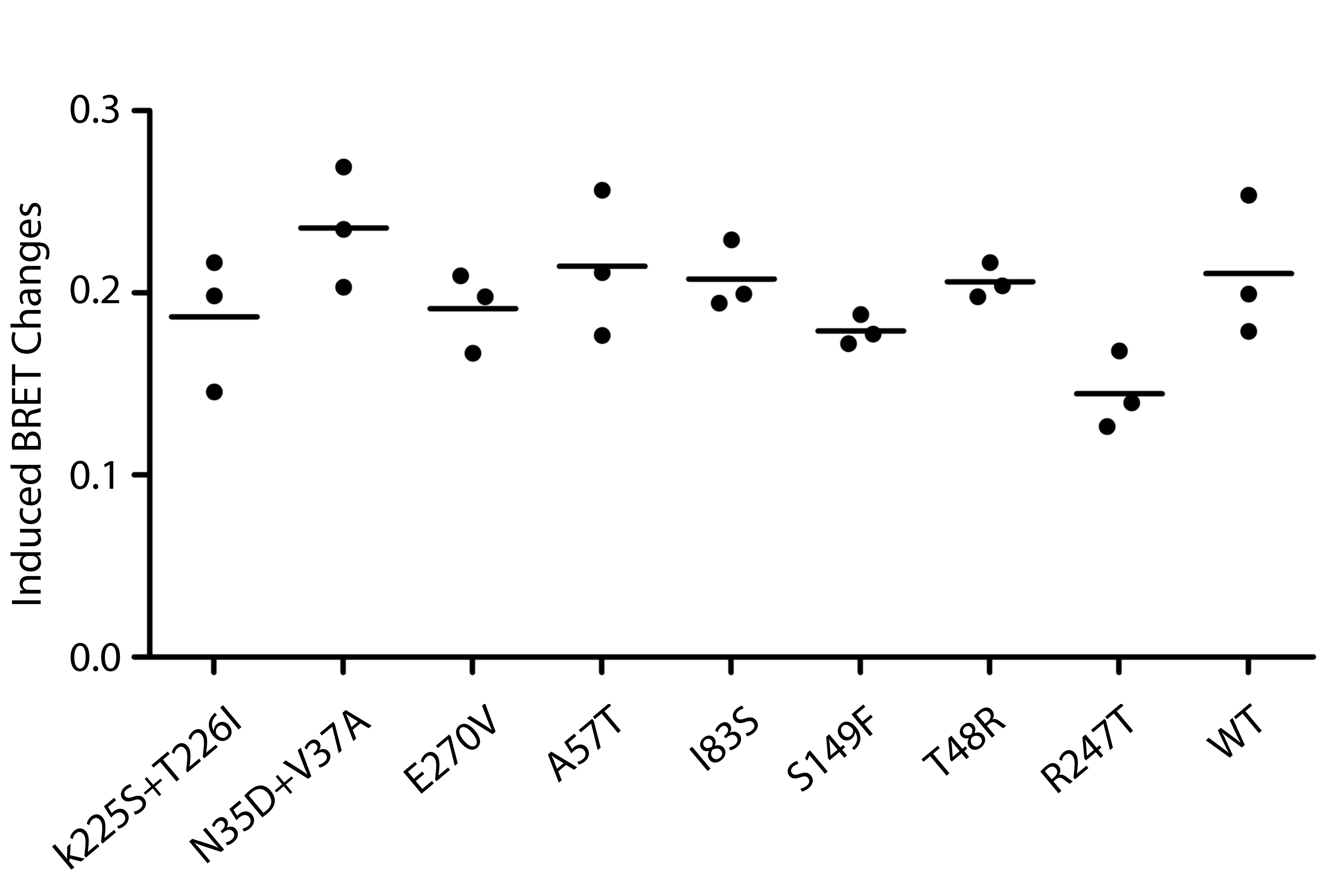

Supplement: Supplementary file 1 [file ijms-22-00151-s001.zip › Supplementary figure 1.tif]

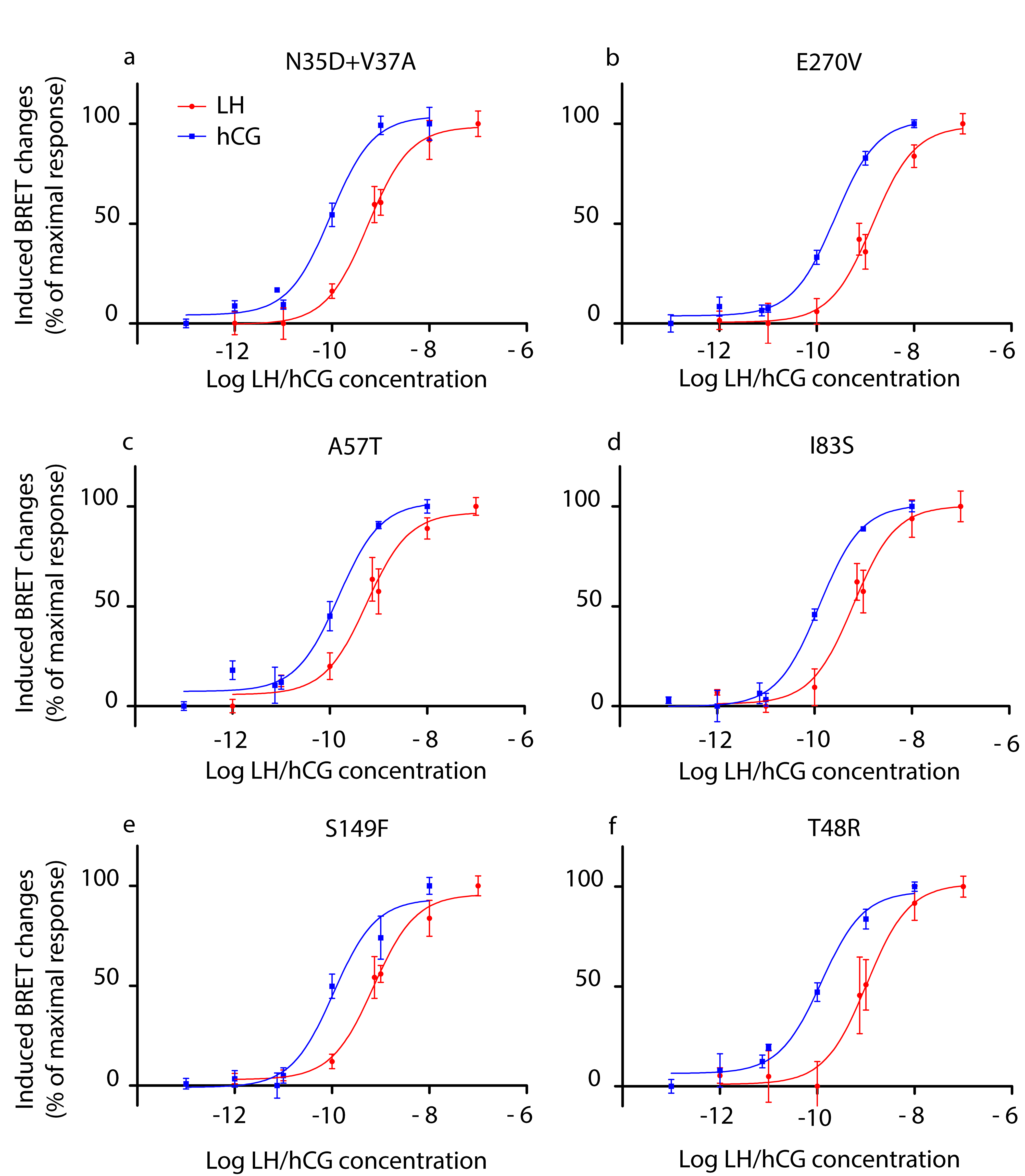

Supplement: Supplementary file 1 [file ijms-22-00151-s001.zip › Supplementary figure 2.tif]

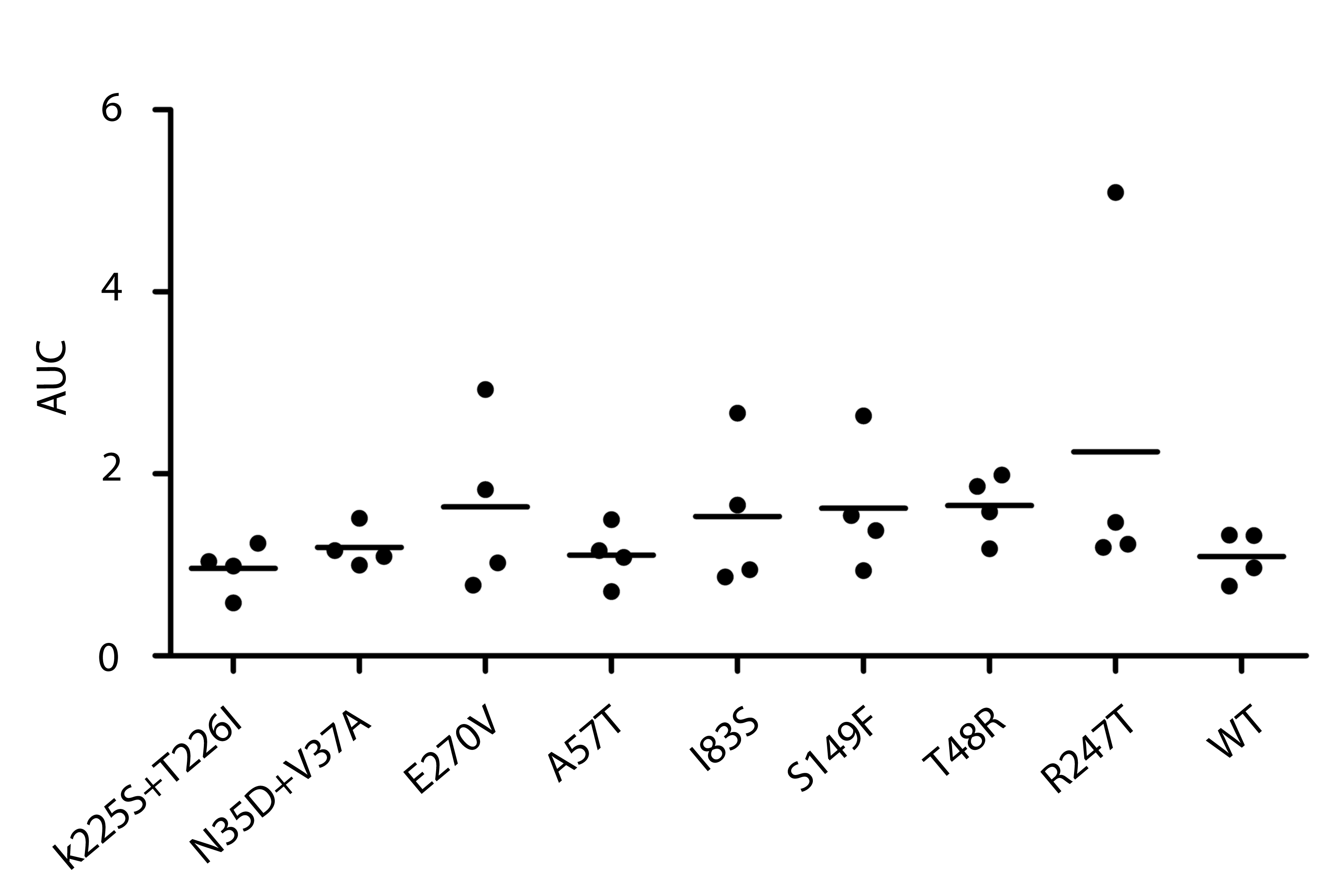

Supplement: Supplementary file 1 [file ijms-22-00151-s001.zip › Supplementary figure 3.tif]

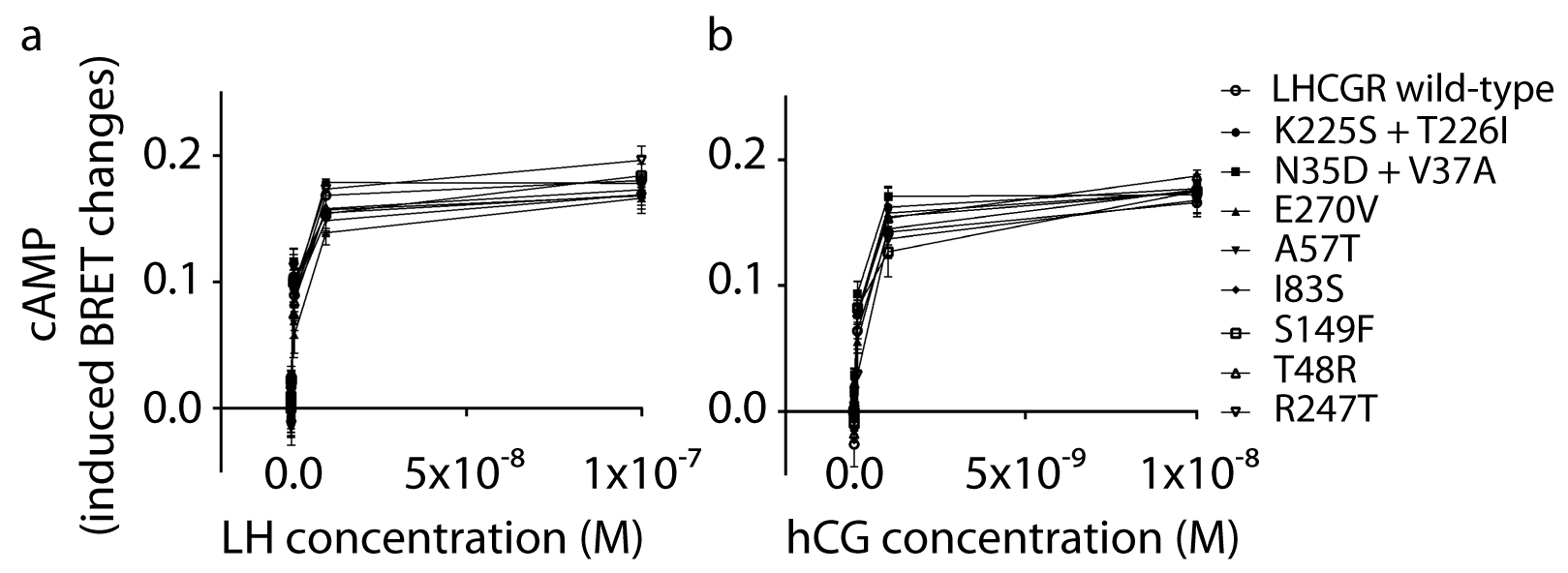

Supplement: Supplementary file 1 [file ijms-22-00151-s001.zip › Supplementary figure 4.tif]
